# Solid Lipid Nanoparticles and Nanostructured Lipid Carriers as Smart Drug Delivery Systems in the Treatment of Glioblastoma Multiforme

**DOI:** 10.3390/pharmaceutics12090860

**Published:** 2020-09-10

**Authors:** Raneem Jnaidi, António José Almeida, Lídia M. Gonçalves

**Affiliations:** Research Institute for Medicines (iMed.ULisboa), Faculty of Pharmacy, Universidade de Lisboa, 1649-003 Lisbon, Portugal; raneemjnaidi@gmail.com (R.J.); aalmeida@ff.ulisboa.pt (A.J.A.)

**Keywords:** glioblastoma multiforme, solid lipid nanoparticles, nanostructured lipid carriers, modification strategies

## Abstract

Glioblastoma multiforme (GBM) is the most common and malignant type of brain tumor. In fact, tumor recurrence usually appears a few months after surgical resection and chemotherapy, mainly due to many factors that make GBM treatment a real challenge, such as tumor location, heterogeneity, presence of the blood-brain barrier (BBB), and others. Solid lipid nanoparticles (SLNs) and nanostructured lipid carriers (NLCs) represent the most promising carriers for therapeutics delivery into the central nervous system (CNS) owing to their inherent ability to cross the BBB. In this review, we present the main challenges in GBM treatment, a description of SLNs and NLCs and their valuable role as drug carriers in GBM treatment, and finally, a detailed description of all modification strategies that aim to change composition of SLNs and NLCs to enhance treatment outcomes. This includes modification of SLNs and NLCs to improve crossing the BBB, reduced GBM cell resistance, target GBM cells selectively minimizing side effects, and modification strategies to enhance SLNs and NLCs nose-to-brain delivery. Finally, future perspectives on their use are also be discussed, to provide insight about all strategies with SLNs and NLCs formulation that could result in drug delivery systems for GBM treatment with highly effective theraputic and minimum undesirable effects.

## 1. Introduction

Glioblastoma multiforme (GBM) is the most common and malignant type of brain tumor in adults. After the initial diagnosis, the median survival of GBM patients is about 12–15 months, even with aggressive treatment [1,2]. Unlike other tumors, GBM treatment represents a major challenge, mainly due to its location in the brain which hinders the complete surgical resection, and the presence of the blood-brain barrier (BBB) that limits drugs entering into the central nervous system (CNS). Despite all the recent advances in the biomedical fields, such as molecular biology, biochemistry, and cell biology, which have led to expanding the insight into the molecular and cellular processes related to GBM development, current treatment is still confined to surgical resection, radiotherapy, and chemotherapy epitomized by temozolomide (TMZ). This standard treatment is applied for newly diagnosed GBM patients and the median survival remains unsatisfactory [3]. However, the development of new drugs has been insufficient to obtain the desired effects in GBM treatment. Most drugs have poor solubility in water, cannot cross the BBB, high doses are required to achieve the effective concentration in the CNS, leading to toxicity consequences and adverse effects. To overcome all these limitations, attempts have been made to develop nanodrug delivery systems which could be the most promising drug carriers in the treatment of GBM [4]. Solid lipid nanoparticles (SLNs) and nanostructured lipid carriers (NLCs), which emerged in the biomedical field 25 years ago, have been tested for the treatment of various diseases such as cardiovascular and cerebrovascular diseases, and have become the standard drug carriers for the latter [5]. SLNs have succeeded in overcoming all the limitations reported with other nanocarriers (niosomes, transfersomes, micelles, liposomes, emulsions, and polymeric nanoparticles) such as toxicity, low loading capacity, and low stability. Moreover, SLN functionalization with a wide range of ligands enables them to deliver treatments specifically to the target tissue. However, SLNs tend to jellify and during recrystallization, no empty spaces are available for the drug which results in drug expulsion [6]. With the aim of improving the entrapment efficiency of SLNs during storage, Müller et al. [7] proposed NLCs as enhanced SLNs through mixing the solid lipids with liquid lipids which created an imperfect crystal structure with more internal space which provided higher entrapment efficiency. This second generation of SLNs improved the entrapment efficiency and also demonstrated more stability properties. The present review provides insights into the importance of solid lipid nanoparticles and nanostructured lipid carriers in GBM treatment, and recent modification strategies in these systems that aim to improve GBM cells targeting, and consequently patients’ outcomes.

## 2. Challenges in the Treatment of Glioblastoma Multiforme (GBM)

**Figure 1:**
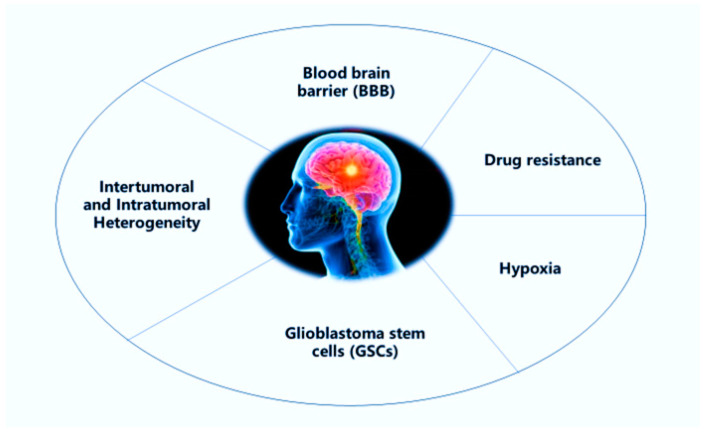
Scheme representing the major challenges in the treatment of glioblastoma multiforme (GBM).

Glioblastoma multiforme (GBM) is the most common and aggressive type of brain tumor, it is also the most fatal human cancer [8]. GBM is responsible for more than 60% of brain tumors in adults, causing premature mortality. The median survival is about 12–14 months, and usually diagnosed patients survive between 1.5–2 years after applying the standard treatment which starts with surgical resection, followed by radiation and chemotherapy [9]. However, various obstacles frustrate the effective treatment of this incurable cancer (Figure 1). Tumor location in the CNS, the presence of BBB, the highly heterogeneous cell population, the invasive nature, glioblastoma stem cells (GSCs), tumor microenvironment, hypoxia, among others, are all factors related to chemoresistance and radioresistance in GBM cells [10].

### 2.1. Blood-Brain Barrier

**Figure 2:**
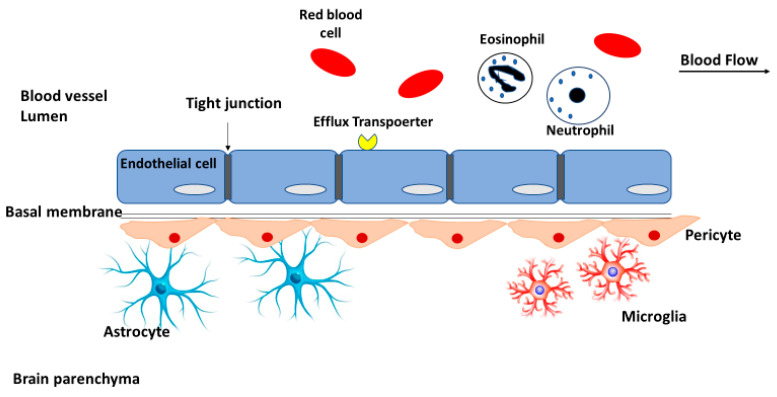
A schematic diagram of the structure of the blood-brain barrier (BBB), the main obstacle for drug penetration into the brain.

The BBB is the main impediment that prevents drugs penetration into the central nervous system to treat GBM and other CNS diseases [11]. It consists of endothelial cells connected by tight junctions (physical barrier). Neurons and glial cells such as astrocytes, microglia, pericytes, and perivascular macrophages also support the structure of the BBB and help to maintain its rigidity [12] (Figure 2).

In addition to all of the structural components that build the physical barrier of the BBB, various drug-metabolizing enzymes are expressed in the BBB and form an enzymatic barrier, including γ-glutamyl transpeptidase (γ-GTP), alkaline phosphatase (AP), and aromatic acid decarboxylase. ATP binding cassette (ABC) proteins, such as P-glycoprotein(P-gp) efflux pumps and multidrug resistance-related proteins (MRPs), are expressed transporters in the BBB that contribute to drug resistance, whereas glucose transporter GLUT1, amino acid carrier LAT1, insulin receptor, and transferrin receptor TfR help to transport drugs into the brain [13]. Since the BBB lacks the presence of paracellular or transcellular channels, drugs can enter into the brain through one of the following two mechanisms: passive transmembrane diffusion and carrier-mediated transport (CMT) or receptor-mediated transport (RMT) [14]. Few molecules can cross the BBB via passive diffusion, providing they have a molecular weight less than 400 Da, lipid solubility, with less than eight hydrogen bonds. In carrier-mediated transport, the drug molecule is, or is synthesized to be, recognized by the influx transport system, without being a substrate of efflux transporters and, consequently, they have high permeability through the BBB. Regarding receptor-mediated transport, large molecules such as peptides and proteins can cross the barrier by binding to receptors that mediate transcytosis from blood to the brain, for instance, TfR receptor and insulin receptor. However, drug molecules could be reengineered with trojan horse molecules that are usually endogenous peptides or monoclonal antibodies (Mab), which cross the BBB using a specific RMT system [15]. Another strategy to transport drug molecules through the BBB is adsorptive-mediated transcytosis (AMT) which takes advantage of the negative charge of the luminal surface of the endothelial cells and provides potential uptake of positively charged molecules such as cationic proteins and peptides from the blood stream to the brain [16]. The concept of AMT was generated by noticing that the polycationic endogenous proteins such as protamine could bind to the endothelial cell surface and cross the BBB [17]. Many strategies have been developed that have enhanced macromolecules penetration into the CNS depending on the AMT process, such as protein cationization which in general involved amidation reactions, cell-penetrating peptides (CPPs) [18], and peptidic transporters that were different in sequence and size (10–27 amino acids residues), such as penetratin and Tat protein [19].

### 2.2. Intertumoral and Intratumoral Heterogeneity

Tumor heterogeneity is a major hallmark that characterizes GBM. The increased incidence of incurable cases, and tumor recurrence after receiving cytotoxic therapy are largely owing to GBM intertumoral and intratumoral heterogeneity which creates a subpopulation of resistant cells. However, recent advances in proteomic and genomic tools have provided a deeper understanding of this heterogeneity that revealed new molecular targets and cell types that were involved in tumor progression [20].

#### 2.2.1. Intertumoral Heterogeneity

Many decades before the clonal evolution, which suggested the vast diversity in cell phenotypes within a tumor, cancer therapies were developed to treat a tumor as a homogenous mass [21]. Recent studies have shown that samples from a recurrent disease shared only 50% of their genetic mutations with primary tumor cells [22]. Intertumoral heterogeneity generated from various factors including differences in the cell of origin, which has been assumed to be a glial stem cell, however, even neurons and mature astrocytes have been proposed to be the GBM cell of origin [23]. The other factors involve genetic and epigenetic alterations that tumor cells are subject to, and lead to hundreds of mutations. According to sequencing and characterization of genome, epigenome, and transcriptome, GBM is classified into three subtypes, i.e., proneural (PN), mesenchymal (MES), and classical (CL). Proneural glioblastoma, which is more common in young adults, has abnormal gene expression of platelet-derived growth factor receptor alpha (PDGFRA), and mutations in the tp53 gene that encodes tumor suppressor protein p53, whereas mesenchymal glioblastoma has an alteration in the gene encoding for neurofibromin (NF1) and classical glioblastoma lacks p53 mutations but has a high level of epidermal growth factor receptor (EGFR) amplification [24].

It has been indicated that traditional chemotherapeutic agents for GBM could also lead to induce mutations and heterogeneity. Treatment with temozolomide (TMZ) is correlated with an increased mutational load in a subset of recurrent TMZ-treated GBM [25]. This intertumoral heterogeneity leads us to conclude that a single therapeutic agent cannot be feasibly effective against all GBM populations. It also highlights the importance of genotyping tumor cells before deciding on the required treatment and shows that single chemotherapy is not efficient for targeting the diverse lesions in GBM.

#### 2.2.2. Intratumoral Heterogeneity

Various studies have defined the heterogenous genetic landscape among GBM patients and suggested that this intratumoral heterogeneity was the key for understanding therapy failure. This heterogeneity has usually been discovered after genotyping the samples from a recurrent tumor. Variation in the methylation status of O^6^-methylguanine DNA methyltransferase (MGMT) promoter has been considered to be the most significant biomarker for predicting the response to chemotherapy. However, this variation was not detected in all patients and other DNA repair mechanisms also exist [10]. Another mutation that contributes to intratumoral heterogeneity in GBM is isocitrate dehydrogenase (IDH1) mutations. The IDH1 gene is mutated in approximately 12% of GBM patients, highlighting that its mutations are detected at only a low frequency in recurrent samples, which indicates the need for temporal sampling [26]. EGFRvIII mutations are one of the most common aberrations that occur in the late stage of GBM and cannot be detected at the initial resection, which makes the use of EGFR-targeted therapy more complicated [27]. Furthermore, intratumoral heterogeneity was noticed through platelet-derived growth factor receptor α (PDGFR α) amplification, which led to defining GBM subpopulations with a variant response to growth factor [28]. All these aberrations have created multiple clonal subpopulations, highlighting the importance of personalized medicine in overcoming inter- and intratumoral heterogeneity in GBM [29].

### 2.3. Glioblastoma Stem Cells

Glioblastoma stem cells (GSCs) are slow-dividing cells, which is only a small subpopulation within the heterogeneous cell populations in GBM. GSCs have the ability to self-renew and their division results in differentiation into two heterogeneous GBM cells or self-renewal GSCs [30]. They are recognized as a potential source for resistance to chemotherapy and tumor recurrence, usually occupying the perivascular niches surrounding blood vessels within the tumor, and they are generally leaky and fragile. Therefore, chemotherapy passage into tumor mass is poor [31]. In addition, GSCs are frequently found in hypoxic regions where GSCs are prominent factors for neo-angiogenesis. Through intercellular signaling, GSCs prompt tumor-associated endothelial cell proliferation, growth, and tube formation which contribute to tumor vascularization [32]. Furthermore, GSCs serve in GBM cell evasion from the immune system by inhibiting T-cell proliferation and activation and inducing apoptosis [33]. To isolate GSCs, CD133, a transmembrane glycoprotein, is the most reliable biomarker. However, other biomarkers have been identified such as CD15, CD44, CD90, and Notch [34,35]. The molecular mechanisms by which GSCs resist chemo- and radiotherapy involve the following: increasing DNA repair capacity, changing cell cycle regulation, and impediment drug accumulation into tumor cells. For instance, GSCs overexpress the O^6^-methylguanine DNA methyltransferase (MGMT) gene which repairs DNA damage caused by alkylating agents such as TMZ. Another example of a resistance mechanism is that ATP-binding cassette transporter protein, ABCG2, is highly expressed in GSCs and it is associated with multidrug resistance and efflux of drugs out of tumor cells. Inhibition of ABCG2 gene using small molecules such as miRNA-328 could be a promising strategy to enhance the potency of chemotherapeutic agents [36].

### 2.4. Drug Efflux

Drugs are usually transported into GBM cells using ATP-dependent proteins, according to the concentration gradient. Those ATP-dependent proteins are represented by the ATP-binding cassette (ABC) transporter family. The ABC family is considered to be one of the main hotbeds of resistance against drug delivery into GBM cells and includes P-glycoprotein (P-gp/ABCB1), multidrug resistance protein (MRP/ABCC2), and breast cancer resistance protein (BCRP/ABCG2) [37]. Erlotinib is an inhibitor of epidermal growth factor receptor (EGFR) tyrosine kinase and it is a substrate of P-gp and BCRP. It has been shown that P-gp and BCRP collaborate in restricting erlotinib penetration into the intracranial tumor and that using strategies to inhibit these efflux pumps could enhance the delivery of substrate drugs such as erlotinib, and others [38]. P-gp is also implicated in chemoresistance to TMZ treatment. TMZ competes with other P-gp substrates which highlights the promising strategy of combined targeted therapy in GBM [39].

### 2.5. Hypoxia

The accelerated rate of GBM cell proliferation results in creating regions with insufficient blood supply. In the end, chemotherapeutic drugs could kill cells on the exterior part of the tumor mass, while the core of the tumor mass that contains inactive cells in the acidic hypoxic environment would survive from chemotherapy since it was unreachable [40]. Moreover, most cancer drugs target high proliferative cells, while cells in the hypoxic area are not rapidly dividing. Hypoxia is considered to be a significant concern for GBM patients, since cells in the hypoxic area tend to evade the unfavorable conditions into healthier brain tissue leading to tumor invasion, the major cause of death in GBM [41]. The transcription factor, HIF-1, is a heterodimer complex consisting of α (HIF-1α, HIF-2α and, HIF-3α) and β subunits. The HIF1-α subunit has been recognized as the master driver for several pathways involved in tumor aggressiveness [42]. In normoxic conditions, HIF1-α is targeted by the von Hippel-Lindau (VHL) protein which activates proteasomal-mediated degradation followed by prolyl hydroxylation of the oxygen-dependent domain (ODD). Another regulatory protein is hydroxylating asparagine residues that prevent interaction with the transcriptional coactivators. Hypoxic conditions decrease the activity of prolyl hydroxylases (PHD 1, 2, and 3) enzymes, resulting in decreased hydroxylation which finally leads to HIF1-α accumulation in the cytoplasm. Afterward, it translocates into the nucleus and interacts with HIF1B and the resulted heterodimer recognizes and binds to hypoxia response elements (HRE) on the promoter region of the genes involved in angiogenesis, glucose metabolism, and cell survival [43]. HIF-1 inhibition has sensitized glioma cells to TMZ through the downregulation of the expression of MGMT gene [44]. Moreover, studies of hypoxia-mediated chemoresistance have found that hypoxic cells upregulated ABCB1 expression and P-gp function which decreased response to chemotherapy [45]. One of the strategies that was developed to target hypoxic areas was to use drugs that were toxic only in acidic hypoxic conditions. Tirapazamine is an experimental anticancer drug that gives a toxic radical only in poorly oxygenated cells, therefore, it kills tumor hypoxic cells without affecting normal tissues. However, tirapazamine failed to prove a significant difference in survival advantage in a group of population treated with tirapazamine and radiotherapy as compared with a control group [46].

## 3. Solid Lipid Nanoparticles (SLNs) and Nanostructured Lipid Carriers (NLCs) for Targeting Glioblastoma

All the mentioned factors contribute to make GBM treatment a real challenge. In particular, due to the presence of BBB that hurdles drug access into the CNS, there is an urgent need to develop efficient drug delivery systems that are able to improve drug concentration in the brain for the treatment of oncologic and neurodegenerative diseases. One of the promising approaches is the use of lipid nanoparticles (LNPs) which have been developed to overcome all the limitations related to polymeric nanoparticles such as high production cost, high toxicity due to use of solvents in their fabrication, and sometimes an allergy to the polymer [47]. The first traditional model of lipid-based nanoparticles was liposomes, which were introduced into the biomedical field in 1965. The first nanomedicine used in human medicine was Ambisome^®^ (Nexstar, San Dimas, CA, USA), a liposomal nanoformulation of amphotericin B, approved in 1990, in Europe. A few years later (1995), the PEGylated liposomal formulation of doxorubicin Doxil^®^ (Alza, Mountain View, CA, USA)/Caelyx^®^ (Janssen-Cilag, Europe) was approved by the FDA for cancer treatment. Despite the unique advantages of liposomes such as high biocompatibility, low toxicity, non-immunogenicity, and biodegradability their applications have been limited due to some related disadvantages. For instance, phospholipids in liposomes can suffer oxidation and hydrolysis reactions, poor stability, short shelf life, low encapsulation efficiency, and high production cost [48]. In the 1990s, SLNs and NLCs were identified as a substitute drug carrier to classical nanocarriers, such as polymeric nanoparticles liposomes and emulsions. These LNPs are spherical in shape and can be produced using physiological lipids or other lipids that have been proven to be safe for use in humans. SLNs and NLCs have demonstrated higher stability and better profile release as compared with liposomes, and a better safety profile as compared with polymeric nanoparticles based on not using organic solvents [49,50,51]. SLNs and NLCs promote drug delivery into the targeted cells through various mechanisms which include active and passive targeting. In passive mechanisms, SLNs and NLCs take advantage of specific properties in the tumor microenvironment to improve drug delivery based on what is called enhanced permeability and retention effect (EPR). However, in active mechanisms, SLNs and NLCs surface is modified to recognize a transporter or a receptor that is overexpressed in the target cells, and this can lead sometimes to selective targeting and minimize side effects. Furthermore, SLNs and NLCs have the inherent ability to cross the BBB and are suitable carriers for a wide spectrum of GBM treatments such as large molecules, genes, oligonucleotides, siRNA, and enzymes. All these characteristics make SLNs and NLCs one of the great candidates as drug carriers in the treatment of GBM and other brain diseases [52]. Although these nanoparticles show all these advantages, SLNs have some disadvantages such as an unexpected tendency to gelation, low encapsulation efficiency (EE), and unpredictable expulsion of the incorporated therapeutic due to solid lipid recrystallization which makes it difficult to keep drug trapped. These limitations were the main reason behind the idea of introducing a liquid lipid into SLN formulation and creating the so-called NLC [50].

### 3.1. SLN and NLC as Smart Drug Delivery Systems in the Treatment of GBM

**Figure 3:**
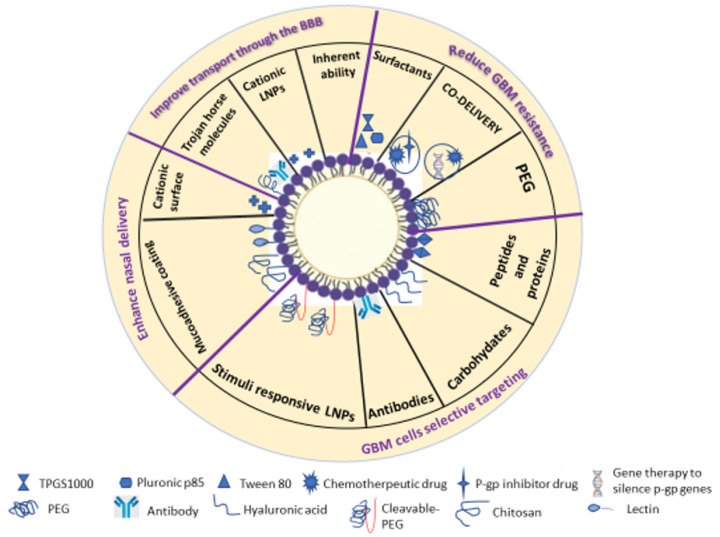
Modification strategies of solid lipid nanoparticles (SLNs) and nanostructured lipid carriers (NLCs) to enhance GBM treatment.

During the last years, various approaches have been developed to create a versatile nanoplatform. Thus, different strategies for designing nanoparticles have been tested to convert simple nanoparticles for drug delivery to the body without considering many issues related to smart nanocarriers with optimized characteristics. A smart drug delivery system is a carrier that can deliver drugs or therapeutics to the target cells without affecting normal tissues, has a specific desired release profile, can evade immune system cleansing, and finally can be used for co-delivery of drug with another substance such as genetic material, diagnosis agents or, in some cases, for combined chemotherapy [53]. Regarding SLNs and NLCs, various modifications have been used to transform them from conventional drug carriers into smart drug carriers which can overcome all the barriers and challenges of GBM treatment (Figure 3).

### 3.2. Modification Strategies to Enhance Crossing the Blood-Brain Barrier (BBB) in GBM Treatment

BBB is the first critical biological obstacle to effective GBM treatment. As mentioned above, SLNs and NLCs have some ability to cross the BBB due to their lipidic nature. In general, this type of strategy often takes advantage of two BBB features, i.e., the RMT system route using trojan-horse molecules attached to nanocarrier or the AMT system route using cationic nanoparticles [54,55]. Many studies have reported that conjugating angiopep-2 on the surface of nanoparticles enhanced drug delivery into GBM cells, since it could bind to lipoprotein receptor-related protein 1 (LRP1) on the BBB. Kadari A et al. [56] studied angiopep-2 conjugated SLNs for docetaxel delivery, and demonstrated enhanced permeability through the BBB and increased cytotoxic effect as compared with unconjugated SLNs. In another study, SLNs loaded with etoposide were conjugated with melanotransferrin antibody (MA) for the treatment of GBM. Melanotransferrin is a syaloglycoprotein expressed in the endothelial cells of the BBB and tumor cells, and it plays a role in iron uptake. It was found that it had a new function in transcytosis through the BBB. Melanotransferrin antibody etoposide-loaded SLNs (MA-ETP-SLNs) had tolerable toxicity to endothelial cells and augmented transport with an improved inhibitory effect on GBM cells [57]. Regarding the use of the AMT system to enhance permeability through the BBB, many approaches can be used to develop positively charged LNPs. This includes using cationized proteins such as albumin, cationic lipids like stearylamine, and cell-penetrating peptides (CPP) such as protamine [58]. In a study related to cationic LNPs synthesis, positively charged SLNs were prepared using 3beta-[*N*-(*N*’,*N*´-dimethylaminoethane) carbamoyl] cholesterol (DC-Cholestrol) and conjugated with TfR monoclonal antibody (OX26) to improve baicalin delivery to the brain. It was reported that the positive surface charge of SLNs influenced vascular uptake and with OX26 conjugation they provided higher bioavailability of baicalin in the cerebrospinal fluid [59]. Other authors have innovated carmustine-loaded cationic SLN, functionalized with anti-EGFR against malignant GBM cells. They used the microemulsion method and the cationic surfactant hexadecyltrimethylammonium bromide (HTMAB) to prepare the cationic SLNs, and showed that the smallest particle size, the highest entrapment efficiency, mild toxicity to the BBB endothelial cells, and the lowest release of tumor necrosis factor α (TNF-α) were obtained with a concentration of 1 mM of the cationic surfactant [60]. To deliver doxorubicin into the brain for GBM chemotherapy, another group presented SLNs conjugated with aprotinin and melanotransferrin antibody with the aim of promoting BBB crossing. Aprotinin is a single chain polypeptide which binds specifically with high affinity to low-density lipoprotein receptor (LDLR) related protein (LRP). LRP and melanotransferrin are expressed by human brain microvascular endothelial cells (HBMECs) and U87MG cells, which have been isolated from the brain of GBM patients. Thus, the combination of SLN with aprotinin and anti-melanotransferrin to carry doxorubicin could show interesting results. They found that increasing 1,2-dipalmitoyl-*sn*-glycero-3-phosphocholine (DPPC) weight percentage promoted zeta potential absolute value and decreased the entrapment efficiency of loaded doxorubicin. This functionalized nanocarrier system provided a sustained drug release profile for doxorubicin and, although it had some toxicity to HBMECs, it improved permeability through the BBB and reduced malignant U87MG cells viability [61]. Agarwal et al. [62] exploited cationic bovine serum albumin (CBSA), which accumulated in the brain, as a ligand conjugated to methotrexate-loaded SLNs to increase transport across the BBB. SLNs were prepared using the melt-dispersion technique followed by ultrasonication. The carboxylic group of SLN was conjugated to the amino groups in CBSA according to a reported process. A cellular uptake study was performed using brain endothelial cells and HNGC1 cell line and measured via flow cytometry and the highest cellular uptake was noticed with CBSA-conjugated SLNs in both cell lines. Regarding the cytotoxicity study, the conjugation with CBSA led to a significant increase in cytotoxic response, and HNGC1 cells were more sensitive to CBSA-conjugated SLNs as compared with free methotrexate and unconjugated SLNs. The authors concluded that CBSA had triggered the AMT route, thus providing better internalization.

### 3.3. Modification Strategies Against GBM Cell Resistance

Multidrug resistance (MDR) is the main obstacle to attain successful chemotherapy in GBM treatment. MDR is usually mediated by three P-gp, BCRP, and MRP-1. Various nonionic surfactants have shown the ability to reverse MDR mechanisms. For instance, Pluronic P85 (a block copolymer surfactant) can sensitize MDR tumor cells corresponding to various chemotherapeutic agents through ATP-depletion [63]. Furthermore, Brij nonionic surfactants have also shown an inhibitory effect against P-gp efflux pumps via the same mechanism. Another nonionic surfactant to bypass P-gp effect is TPGS 1000 (d-alpha-tocopheryl polyethylene glycol 1000 succinate) that can alter efflux transport activity through ATP inhibition without being a P-gp substrate or competitive inhibitor [64]. One group has fabricated Compritol^®^ (Gattefossé, Lyon, France) and Precirol^®^ (Gattefossé, Lyon, France) lipid nanoparticles coated with Tween^®^ 80 (Roig Farma, Barcelona, Spain) as a surfactant and P-gp inhibitor. Those lipid nanoparticles that were loaded with edelfosine were tested in C6 glioma cell line and the same cell line was used to create an in vivo xenograft mice model of glioma. Their results demonstrated increased accumulation of edelfosine in the brain due to Tween^®^ 80 coating and P-gp efflux inhibition [65]. In another study, TPGS-SLNs were used to enhance *trans*-resveratrol (RSV) passive brain targeting. TPGS-RSV-SLNs were prepared using the solvent emulsification evaporation method and the optimized formulation showed an average particle size of 203.1 ± 14.91 nm and a polydispersity index value of 0.263 ± 0.12. A biodistribution study for free RSV and TPGS-RSV-SLNs, in two groups of rats, revealed that the optimized formulation showed a brain accumulation 9.23 times higher than that of RSV solution. These results strongly recommended TPGS-SLN to be a suitable carrier to deliver RSV in glioma treatment [66]. Polyethylene glycol (PEG) derivatives have also been proposed as P-gp efflux inhibitors such as PEG stearate and PEG glyceryl fatty acid esters [67]. A previous research, which aimed at providing better penetration and retention of noscapine into the brain for GBM treatment, reported the use of PEG-conjugated SLNs as a suitable approach. Stearic acid, egg phosphatidylcholine, and sodium glycocholate were used as solid lipid, surfactant, and co-surfactant, respectively, and then were coated with PEG stearate. The obtained nanoparticles were less than 100 nm in size with an encapsulation efficiency of 83.6 ± 1.2%. Surface modification with PEG resulted in improved pharmacokinetics and brain delivery and, due to the ionic interaction between the core and coat, a slow drug release was achieved [68]. One of the successful approaches for targeting efflux has been to combine multiple strategies in one nanocarrier. Tang et al. [64] presented SLNs loaded with curcumin (Cur) and piperine (Pip) as co-delivery systems to reverse the MDR effect. TPGS and Brij 78 were used to inhibit drug efflux mediated by P-gp pumps and sensitize tumor cells to anticancer therapy. Additionally, Cur has proven efficacy in downregulating intracellular levels of ABC transporters family including P-gp, MRP-1, and ABCG2. Pip also downregulates the expression of P-gp, MRP-1, and ABCG2 genes. However, despite the pharmacological activity and safety properties of Cur and Pip, their therapeutic use is limited due to their low solubility in water, which leads to low bioavailability, and this impedes incorporating these compounds into a suitable carrier such as nanoparticles to obtain the required systemic concentration. Pip-Cur-SLNs were prepared using emulsification evaporation–low temperature solidification method. To predict the physical stability of the formulations, zeta potential values were measured and Pip-Cur-SLNs showed a high negative value of −20 mV. The mean particle size was 130.8 nm, with PDI less than 0.5, which indicated poor particle aggregation. To assess whether Pip-Cur-SLNs inhibited P-gp function, Rh efflux assay was used. Rhodamin 123 (Rh 123) is a fluorescent dye and a substrate for P-gp, thus, P-gp inhibition correlates with Rh 123 increased intracellular accumulation. A2780/Taxol cells treated with Pip-Cur-SLNs exhibited the highest cellular uptake of Rh123 as compared with free Rh123, free Cur, and free Pip and they also resulted in considerable cytotoxic effect in the same drug-resistant A2780/Taxol cells. Other strategies for inhibiting efflux effect have used SLNs and NLCs for co-delivery of two drugs simultaneously, i.e., a drug efflux inhibitor and a chemotherapeutic agent or gene therapy for efflux gene silencing with a chemotherapeutic agent. Since P-gp efflux restricts docetaxel entry into the brain for cancer treatment, folic acid-modified SLNs were developed to deliver docetaxel and ketoconazole (P-gp inhibitor) for brain targeting [69]. The reported SLNs were evaluated in brain endothelial cells and results revealed that folate modified docetaxel and ketoconazole SLNs had a brain permeation coefficient 44 times higher than that of Taxotere^®^ (Sanofi-Aventis, Bridgewater, NJ, USA).

P-gp gene silencing is another strategy to improve the cellular delivery of drugs. There are only a limited number of studies that have been published on using SLNs and NLCs for the delivery of gene therapy in the treatment of multidrug-resistant tumors and most recent studies have focused on using inorganic or polymeric nanoparticles as a carrier [70]. However, Saad et al. [71] used cationic liposomes as a carrier for doxorubicin and two siRNA, siMRP1 and siBcl-2. Positively charged 1,2-dioleoyl-3-trimethylammonium-propane (DOTAP) was used to prepare the cationic liposomes through an ethanol-injection method and they were loaded with negatively charged siRNA via electrostatic interaction. Empty cationic liposomes did not cause a significant change in the expression of the targeted mRNA, whereas opposite results were obtained after the incubation with liposomes containing doxorubicin and siRNA. In fact, treating cells with free doxorubicin led to MRP1 and BCL2 mRNA overexpression and these results supported the data that chemotherapy stimulated resistance mechanisms in tumor cells. The simultaneous carrier could suppress two types of resistance by delivering anticancer drugs at the same time which provided high levels of efficacy and enhanced chemotherapy toxicity.

### 3.4. Strategies for Selective Targeting of GBM Cells

Over the last decades, lipid-based nanoparticles have been widely employed to overcome the problems related to conventional chemotherapy which have lacked specificity and led to a spectrum of side effects. Recent efforts have focused on lipid-based nanocarrier surface manipulation through functionalization with various ligands such as peptides, proteins, carbohydrates, monoclonal antibodies, and small molecules. These ligands can recognize overexpressed targets on the surface of tumor cells, and thus result in nanocarrier accumulation in a tumor microenvironment and minimize the unspecific distribution within the body. Other modification strategies have exploited tumor microenvironment characteristics such as acidic pH, hypoxia, and enzymatic hydrolysis through the development of stimuli-sensitive lipid-based nanoparticles [72]. Folate is one of the commonly used small molecules as a ligand for targeted therapy, since the folate receptor is highly expressed in various types of cancer cells such as lung cancer, colon cancer, and GBM. Zhang et al. [73] developed NLCs decorated with folate targeted delivery of etoposide (ETP) (FA-ETP-NLCs). In vitro cytotoxicity of FA-ETP-NLCs was investigated in three cell lines, i.e., CT26, SGC7901, and NCI-H209, and had IC_50_ values three to four times higher than ETP-NLCs which illustrated higher suppression efficacy after targeting with folate. Another group used folic acid and ρ-aminophenyl-α-d-manno-pyranoside (APMP) as ligands conjugated to ETP-loaded SLNs for targeted therapy of GBM. This nanocarrier was able to cross the BBB through glucose transporter 1 and it could better recognize U87MG cells via folate receptors. The APMP-FA-ETP-SLNs demonstrated sustained release with improved-ETP efficacy. Consequently, the effective dosage of ETP against malignant glioma cells could be reduced [74]. Cetuximab is a monoclonal antibody that binds with high affinity to EGFR which is usually upregulated in GBM cells. To obtain more targeted and focused therapy for brain carcinoma, Kuo et al. [60,61] labelled cationic SLNs with anti-EGFR monoclonal antibody (cetuximab) to deliver carmustine into malignant GBM cells. SLNs grafted with anti-EGFR could specifically bind to EGFR in U87MG cells. Thus, this delivery system could enhance carmustine transport to U87MG cells, and therefore reduced the required dose. Peptides also have been emerged as a potent targeting ligand due to their various advantages such as small size, high stability, low immunogenicity, and high selectivity [75]. RGD (arginine-glycine-aspartic acid) is a peptide that is widely used in neovasculature targeting delivery since it binds with high affinity to integrin receptors which are overexpressed on both GBM cells and the endothelial cells of tumor vessels. Song et al. [76] developed RGD-conjugated NLCs for TMZ delivery into GBM cells. The RGD-TMZ-NLCs showed a higher cytotoxic effect on U87MG cells than TMZ-NLCs and they also had a higher antitumor efficiency in vivo. Endogenous proteins can also be used effectively as targeting ligands, since they selectively bind to specific receptors on cell surface and can mediate endocytosis such as transferrin, lactoferrin, and IL-13 [77]. Lactoferrin (Lf) is a cationic iron-binding glycoprotein of the transferrin family. Lactoferrin receptors are expressed on BBB endothelial cells and the surface of GBM cells. To enhance targeting competence of SLNs in brain tumor treatment, Singh et al. [78] prepared Lf-SLNs for proficient delivery of docetaxel into the brain. The targeting mechanism and cellular uptake in tumor cells for Lf-docetaxel SLNs were evaluated through receptor saturation assays and distribution studies in the brain. Lf conjugating on the surface of SLNs improved targeting potency and enhanced the inflow inside the brain. Regarding carbohydrate ligands, hyaluronic acid is a natural polysaccharide that shows good binding affinity for CD44 that is highly expressed on various tumors such as melanoma, breast cancer, colorectal cancer, and brain cancer. Hayward et al. [79] employed hyaluronic acid as an active targeting ligand to develop a liposomal drug delivery system that could distinguish between malignant GBM cells and healthy brain cells. Hyaluronic acid-coated liposomes’ cellular uptake was evaluated in primary astrocytes, microglia, and GBM cells and the system showed selective targeting of tumor cells over normal cells, since GBM cells exhibited higher expression of CD44. Hyaluronic acid has also been applied for targeted delivery of SLNs and NLCs against various tumors [80,81].

Matrix metalloproteinases (MMPs) are a group of proteases enzymes that have elevated levels in GBM cells and an array of tumors. MMPs are present as catalytic markers in the tumor microenvironment and have long been related to cancer cell behaviors such as migration, invasion, apoptosis, and differentiation. Thus, MMP enzymes could be employed as triggers of nanoparticle enzymatic activation [82]. Bruun et al. [83] discussed developing a nanocarrier that showed the lowest cellular interaction during systemic circulation, while it exhibited high uptake from targeted cells. They designed lipid nanoparticles encapsulated with siRNA to target U87MG cells. The targeting delivery was obtained using two strategies. First, the LNPs’ surface was decorated with angiopep that targeted LRP-1 on the BBB, and then those cationic nanoparticles were coated with PEGylated (poly(ethylene glycol)) cleavable lipopeptide that included a substrate for MMPs. Then, dePEGylation of the system by MMPs was followed by a charge shift from negative to positive, which triggered cellular endocytosis and siRNA release.

Owing to the elevated activity of the proton pump and glycolysis in tumor cells, the extracellular pH is slightly more acidic than normal tissues (between 6.5 and 7.2). A pH-sensitive drug carrier can be developed through using pH-labile chemical bonds such as β-carboxylic acid, amide, hydrazone, acetal, orthoester, and glycerol ester groups. These bonds show stability in neutral and alkaline mediums, while they tend to be hydrolyzed under acidic conditions, and enable drug release from the disturbed nanocarrier after linkage degradation [84]. In addition, pH-sensitive PEG-modified nanoparticles have also been developed for targeted treatments. Chuang et al. [85] developed pH-sensitive cationic PEGylated SLNs (PEG-SLN+) for effective targeting and accumulation of camptothecin in tumor cells. They found that PEG-SLN+ could release the drug efficiently in low pH conditions (pH 5.5), whereas it showed stability in pH value of 7.4. In vitro and in vivo studies have revealed selective and long-term accumulation into the cytoplasm of tumor cells. Several studies have highlighted the drawbacks of coating nanocarriers with PEG, namely, so-called “PEG dilemmas”, which indicated interaction hindrance between the carrier and the target cell. To mitigate this problem, Kim et al. [86] utilized a tumor acidic environment to develop RIPL peptide NLCs conjugated with cleavable PEG that had been removed from th nanocarrier surface at the tumor site. Cleavable PEG was prepared using DPPE (1,2-dipalmitoyl-*sn*-glycero-3-phosphothioethanol) as hydrophobic anchor which was linked to PEG3000 chain by hydrazone bond. The results showed that 5 mol% PEG was efficient to control plasma proteins adsorption and minimize macrophages uptake. PEG cleavage was also tested at different pH values that mimic normal or tumor conditions and at pH value of 6.5, and the cellular uptake was increased over two times as compared with PEG-RIPL-NLCs. However, the cellular uptake of cPEG-RIPL-NLCs was less than intact RIPL-NLCs and this was interpreted as the effect of residual PEG on nanoparticles surface.

Hypoxia is a notable feature of solid tumors and the redox potential between normal and tumor tissue is different. This difference can be exploited to design hypoxia-sensitive drug delivery systems. Various studies have shown that glutathione levels are 100–1000-fold higher than blood and 100-fold higher than normal tissues. Thus, the development of nanoparticles that include disulfide bonds could maintain carrier structure under normal conditions. In glutathione-rich cells, these bonds are reduced to thiol groups and the integrity of the nanoparticles would be compromised resulting in the content release [87]. McNeely et al. [88] formulated cysteine-cleavable phospholipid-polyethylene glycol (PEG) liposomes for GBM active targeting. This polymer coating could be removed as liposomes were extravasated to tumor cells, and then targeting ligands were unmasked. Cysteine-cleavable phospholipid PEG masking enabled nanocarriers to evade the reticuloendothelial system (RES) and provided a prolonged circulation time. The formulation was evaluated in 9 L glioma cells and enhanced cellular uptake and cytotoxicity were observed due to cysteine cleavage of disulfide bonds and PEG5000 chains removal.

### 3.5. Modified Lipid Nanoparticles for Nose-to-Brain Delivery

The direct anatomical connection between the nasal cavity and the CNS makes the field of nasal drug delivery into the brain one of the most interesting applications due to various benefits that overcome problems of conventional routes of administration. This includes non-invasiveness, ease of administration, fast onset of action, wide absorption area, reduced enzymatic activity, and evasion of hepatic first-pass metabolism. These various advantages explain the increased number of products, in the market, that exploit the nose for CNS delivery [89]. Among various nanocarriers, LNPs including SLNs and NLCs administered through the nasal route, have proven effectiveness as drug carriers for treating CNS diseases such as neurodegenerative diseases and brain tumors. The mechanism by which nanoparticles improve drug delivery from the nose to the brain is that they interact with the mucus layer, and then release incorporated drug into the mucus cells, or they cross the mucus layer to be taken up by neurons to finally translocate in nerve axons and reach the brain where they release drug. The nanoparticles’ efficient interaction with this biological environment is influenced by the physicochemical characteristics of the carrier such as composition, size, and surface charge. For instance, mucociliary clearance is one of the significant factors that affects drug nose-to-brain delivery by limiting the residence time of substances administered via the nasal cavity [90]. Therefore, coating LNP surfaces with mucoadhesive compounds such as chitosan, hyaluronic acid, or low-molecular weight pectin could prolong residence time in the olfactory region [91]. Furthermore, mucins in the mucus layer contain elevated levels of sialic acid and sulphate residues, which add a negative charge to this layer and contribute to its rigidity. Consequently, using nanoparticles that have been modified to have a cationic surface would prolong their interaction with mucosa due to the electrostatic attraction. However, the prolonged contact of formulation with nasal mucosa could lead to some undesirable side effects such as irritation and epithelial cell toxicity. Protecting primary olfactory nerves and the sense of smell from damage caused by cytotoxic drugs has to be considered [92]. SLNs and NLCs are described as superior candidates for GBM targeting through the nasal route owing to their high biocompatibility, low toxicity, and ease of surface functionalization. Madane et al. [93] developed curcumin-loaded NLCs for intranasal delivery to the CNS. To evaluate carrier nasal permeation, an ex vivo study was conducted using the nasal cavity of sheep and the permeability coefficient had a value of 3.8 cm^2^/min. A biodistribution study in rats displayed a significant increase of curcumin in the brain after nasal administration of Cur-NLCs with *C*_max_ 86,201  ±  8182.1 ng/g at *t*_max_ of 120 min. TMZ-NLCs have also been developed to target GBM via the intranasal route. In vivo studies have demonstrated improved brain accumulation of TMZ-NLCs as compared with free TMZ, which proved the significant efficacy of intranasal administration of NLCs [94]. Wang et al. [95] aimed at enhancing Pueraria flavones brain delivery through the intranasal administration of borneol SLNs. Borneol is a bicyclic monoterpene that has been used widely in Chinese traditional medicine for guiding drugs to the brain. Recent studies have proven that borneol increased transport through the BBB and also improved drug permeability through the nasal mucosa [96]. *Pueraria* flavone-loaded SLNs were prepared using the emulsification evaporation-low temperature solidification method with borneol or without borneol. Cellular uptake was evaluated in Caco-2 cells in vitro and also in rats, to predict brain targeting after nasal delivery. The highest accumulation of SLNs in the brain was achieved with bornoel-stearic acid SLNs, which had a particle size of 160 nm and drug loading of 4.7% with good release and stability properties. One group designed formulations of chitosan-coated NLCs, and obtained positively charged nanoparticles with mucoadhesive properties which improved drug delivery to the brain via the intranasal route. The formulations exhibited prolonged retention time in the nasal epithelium which suggested that they were a promising carrier to reduce drug dose and dosage frequency. However, in vivo fluorescence monitoring results demonstrated lower accumulation of these nanoparticles in the brain as compared with the lungs, which warranted the need for more modifications that could increase the percentage of formulations reaching brain after the nasal delivery [97]. Nanoparticles intended for nasal delivery can also be modified by incorporating lectin to the surface. Lectins are glycoproteins extracted from some plants such as tomatoes and wheat germ, and they have a selective affinity toward glycan residues on biological surfaces. Therefore, lectins could be useful for targeting delivery systems and they have been used to enhance adsorption in the gastrointestinal tract [98]. Previous works by Broadwell and Balin [99] and Thorne et al. [100] have shown that wheat germ agglutinin (WGA) improved accumulation in the olfactory bulb through binding to the surface of olfactory sensor cells and subsequently enhanced nose-to-brain delivery in a qualitative and quantitative way. WGA also binds to N-acetyl-d-glucosamine and sialic acid residues are abundant in the nasal epithelial structure. However, nanoparticles functionalized with lectin for targeting nasal epithelium could be problematic, since lectins have toxicity effects to mammalian cells [101]. Table 1 includes examples of the studies that tested the use of modified SLNs/NLCs to obtain effective drug delivery in the treatment of GBM. 

## 4. Future Perspectives of SLNs and NLCs for Glioblastoma Treatment

The current treatment of GBM is considered to be a major challenge because of the difficulty of drug delivery into the brain, and also due to tumor heterogeneity, resistance, aggressiveness, and invasive nature. SLNs and NLCs as drug carriers for GBM effective treatment is a fascinating scientific topic that provides hope for GBM patients. Despite all the promising results in the literature related to using SLNs and NLCs in GBM treatment with various drugs, different materials, and different approaches of functionalization, to the best of our knowledge, none of these carriers have been successfully developed by a pharmaceutical company and entered the market. Therefore, it is imperative to re-evaluate all the approaches currently used to design SLNs and NLCs. In our opinion, more efforts should be focused on the required techniques for large-scale development or reproducible nanocarriers. Additionally, more studies are also in demand to clarify safety issues of nanoparticles related to their nano-size and the fact that they can pass through cell membranes in the organism and react with various biological systems. This could be studied by carrying out in vivo experiments that would help predict nanoparticles toxicity in all organs. For nanoformulations that are intended for nose-to-brain delivery, a long-term toxicity study in the lungs should be done to confirm their safety. Taking all these problematic issues into account could promise significant advances in using SLNs and NLCs as smart drug delivery systems in the treatment of malignant GBM.

## Figures and Tables

**Table 1:** SLNs and NLCs for enhanced treatment of GBM.

**Strategies to Enhance Crossing the BBB**				
**Formulation**	**Cargo/drug**	**Ligand**	**Target**	**Ref**
SLN	Docetaxel	Angiopep-2	lipoprotein receptor related protein 1 (LRP1)	[56]
SLN	Etoposide	melanotransferrin antibody (MA)	Melanotransferrin	[57]
Cationic SLN	Biacalin	OX26 monoclonal antibody	Transferrin receptor (TfR)	[59]
Cationic SLN	Carmustine	Anti-EGFR	EGFR	[60]
SLN	Doxorubicin	Aprotinin, melanotransferrin antibody	low-density lipoprotein receptor (LDLR) related protein (LRP), melanotransferrin	[61]
SLN	Methotrexate	Bovine serum albumin (BSA)	Negative charge of BBB endothelial cells membrane	[62]
**Modification Strategies Against GBM Cells Resistance**				
**Formulation**	**Cargo/drug**	**Strategy**	**Target**	**Ref**
SLN	Edelfosine	Tween^®^ 80	P-gp efflux	[65]
SLN	Trans-Resveratrol	TPGS	P-gp efflux	[66]
SLN	Noscapine	PEG	P-gp efflux	[68]
SLN	Curcumin, Piperine	TPGS and Brij 78	MDR effect	[64]
Folate SLN	Docetaxel	Ketoconazol	P-gp efflux	[69]
**Strategies for Selective Targeting of GBM Cells**				
NLC	Etoposide	Folic acid	Folate receptor	[73]
NLC	Etoposide	Folic acid, ρ-aminophenyl-α-d-manno-pyranoside (APMP)	Folate receptor, glucose transporter 1	[74]
SLN	Carmustine	Cetuximab	EGFR	[60]
NLC	Temozolomide	RGD peptide	Integrin receptors	[76]
SLN	Docetaxel	Lactoferrin	Lactoferrin receptors	[78]
SLN	Vorinostat	Hyaluronic acid	CD44	[81]
LNP	siRNA	PEGylated (poly (ethylene glycol)) cleavable lipopeptide	MMPs	[83]
Cationic SLN	camptothecin	Cleavable PEG	Tumor low pH	[85]
**Modified Lipid Nanoparticles for Nose-to-Brain Delivery**				
SLN	Pueraria flavone	Borneol	Improve crossing the BBB and permeability through nasal mucosa	[95]
NLC	Proteins	Chitosan	Prolonged interact with nasal mucosa	[97]
